# Nitrogen-doped hollow carbon spheres with tunable shell thickness for high-performance supercapacitors†

**DOI:** 10.1039/d0ra02935a

**Published:** 2020-07-14

**Authors:** Dawei Zhang, Shaodian Shen, Xiuzhen Xiao, Dongsen Mao, Baoman Yan

**Affiliations:** Research Institute of Applied Catalysis, School of Chemical and Environmental Engineering, Shanghai Institute of Technology Shanghai 201418 China 13855160087@163.com 18355109981@163.com +86-21-64252923; Key Laboratory for Advanced Materials and Research Institute of Industrial Catalysis, East China University of Science and Technology Shanghai 200237 China; Shanghai Institute of Science and Technology China

## Abstract

Nitrogen-doped hollow carbon spheres (NHCSs) are well prepared by using Cu_2_O microspheres as a hard template and 3-aminophenol formaldehyde resin polymer as carbon and nitrogen precursors. The thickness of the carbon shell can be easily controlled in the range of 15–84 nm by simply adjusting the weight ratios of the precursors to Cu_2_O microspheres, and the Cu_2_O templates can also be further reused. Physicochemical characterization demonstrates that the obtained NHCSs possess a well-developed hollow spherical structure, thin carbon shell and high nitrogen doping content. Due to these characteristics, when further utilized as electrodes for supercapacitors, the NHCSs with the carbon shell thickness of 15 nm show a high capacitance of 263.6 F g^−1^ at 0.5 A g^−1^, an outstanding rate performance of 122 F g^−1^ at 20 A g^−1^ and an excellent long-term cycling stability with only 9.8% loss after 1000 cycles at 5 A g^−1^ in 6 M KOH aqueous electrolyte. This finding may push forward the development of carbon materials, exhibiting huge potential for electrochemical energy storage applications.

## Abstract 2

Nitrogen-doped hollow carbon spheres (NHCSs) are well prepared by using Cu_2_O microspheres as a hard template and 3-aminophenol formaldehyde resin polymer as carbon and nitrogen precursors.

## Graphical abstract

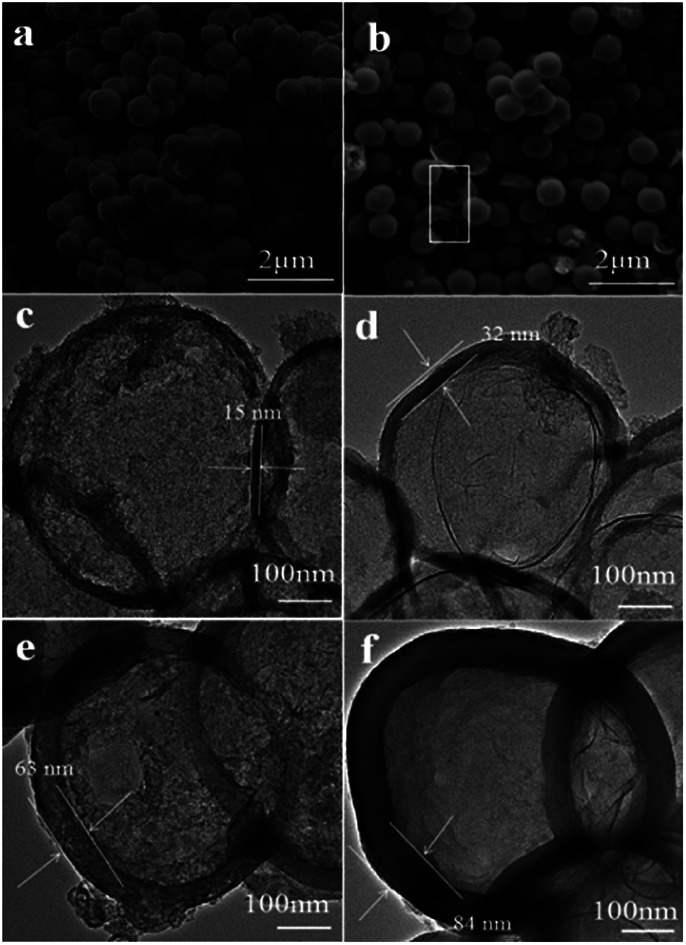

## 1. Introduction

Hollow carbon spheres (HCSs) have been attracting significant scientific attention due to their unique structure and excellent performance, such as very high specific surface, good mechanical flexibility, outstanding chemical stability and high conductivity.^1–3^ These remarkable properties enable them to be widely applied in the field of carriers for drugs, catalysis, fuel batteries and supercapacitors.^4–8^ In particular, HCSs are beneficial in the application of supercapacitors because of their high conductivity and transport lengths for mass and charge transport.^3,9^ In addition, research shows that doping of heteroatoms, such as nitrogen (N) phosphorus (P), boron (B) and sulfur (S), has attracted plenty of attention as an effective way to improve the electrochemical performance by the faradic process generated from the additional electrochemical active sites.^10–14^ Specifically, it is well known that the nitrogen-doped carbon spheres (NHCSs) materials can enhance the capacitance of the material by improving their electronic conductivity and surface wettability.^15^ Currently, a great many of research efforts have been devoted to NHCSs-based electrolyte materials for supercapacitors.

NHCSs have been prepared by means of various synthetic approaches, including the Chemical Vapor Deposition (CVD), electric arc and template methods.^16–22^ Among them, the template method has attracted people's attention due to its advantages such as simple operation and low cost. Template method can be divided into two types: soft-template method and hard-template method. Based on the co-self-assembly of carbon precursors and decomposable amphiphilic molecules, the soft template process is quite easy, while the morphology controllability is relatively unsatisfied.^23^ The hard templates are commonly used to synthesize homogeneous NHCSs by coating the spherical template core with carbon precursors, followed by carbonization and subsequent template etching by acids or organic solvents.^15,24^ Qu^25^ has developed a facile modified template method to synthesize NHCSs in the presence of resorcinol/formaldehyde as carbon precursors and ethylenediamine as both a base catalyst and nitrogen precursor, the obtained NHCSs exhibit a large specific surface area and unique hollow structure. Therefore, the hard template method is simple and effective way to prepare NHCSs. However, the templates currently used are mainly SiO_2_ nanoparticles and polymer latex particles,^26,27^ these templates are non-recyclable, which increases the cost of hard template method to synthesize NHCSs in some certain cases. Thence, we propose a method that the template can be used again, *i.e.* Cu_2_O used as a hard template. Remarkably, the Cu_2_O template can be recycled by the evaporation and crystallization of final waste liquid and followed by reduction for repeated production of NHCSs. It makes the process highly cost-effective and environmentally benign for large-scale production, especially while combining with cheap precursors. Moreover, the thickness of carbon shell can be well controlled. It is realized that the synthesis with an adjustable thickness of carbon shell can effectively improve the dielectric loss characteristics and increase matching characteristic impedance of NHCSs, resulting in enhancing electrochemical performances. However, there are few reports about NHCSs materials with tunable shell thickness in supercapacitors.

**Scheme 1:**
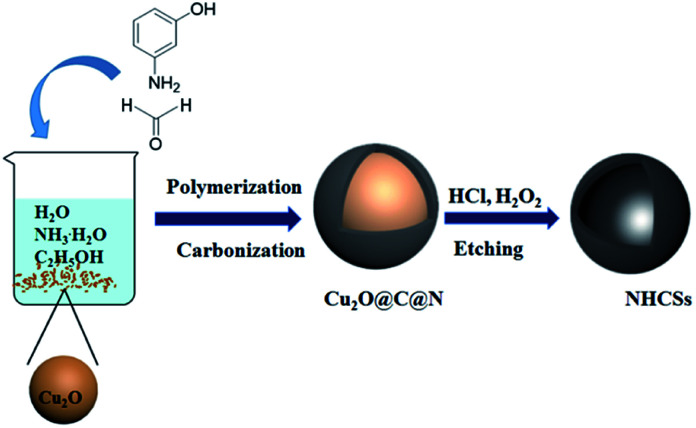
Illustration of the synthesis process of N-HCSs.

Herein, we present a simple and effective strategy to synthesize NHCSs with the well-preserved spherical morphology, carbon shell thickness-controllable and high-nitrogen content *via* a hard template method (Scheme 1). The fabrication process uses 3-aminophenol and formaldehyde polymer resins as carbon and nitrogen precursor, and Cu_2_O solid spheres as hard template. Moreover, the shell thicknesses of NHCSs can be adjusted by changing the ratio of 3-aminophenol to Cu_2_O. Meanwhile the obtained materials reveal fully composed of spherical particles with the diameter of 600–700 nm and shell thickness can be easily controlled in the range of 15–84 nm. In particular, NHCSs with the thinnest carbon shell as supercapacitor electrodes exhibit a high specific capacitance of 263.6 F g^−1^ at a current density of 0.5 A g^−1^, and long-cycling experiments present good cycle stability in 6 M KOH aqueous solution.

## 2. Expermental

### 2.1. Synthesis of Cu_2_O template

Cu_2_O solid spheres with the diameter of 600–700 nm were synthesized *via* a solution-phase method. For synthesis of the spheres, 50 ml of 0.4 M CuCl_2_·2H_2_O solution was mixed with 100 ml of 1 M NaOH and 0.1 g of cetyltrimethylammonium bromide (CTAB) and stirred at room temperature to form a uniform solution. Then, 60 ml of 0.5 M glucose solution and 15 ml of 1 M N_2_H_4_ solution were then added successively to the above solution by strong stirring. During this process, the color of the solution changed gradually into brick-red indicating the formation of the yellow precipitate of cuprous oxide (Cu_2_O), the precipitate was collected by centrifugation and washed sequentially with distilled water and ethanol several time, then dried at 60 °C for 5 h under vacuum.

### 2.2. Synthesis of N-doped hollow carbon spheres (NHCSs)

Nitrogen-doped hollow carbon spheres (NHCSs) were synthesized by using Cu_2_O microspheres as hard template, 3-aminophenol formaldehyde resin polymer as carbon and nitrogen precursors. The synthesis mechanism is illustrated in Scheme 1. Firstly, 1.5 g of Cu_2_O microspheres were dispersed in a stock solution, which contained 80 ml of water, 32 ml of absolute ethanol, and 0.4 ml of ammonia, then the mixed solution was stirred at room temperature for more than 1 h. Subsequently, 0.1 g of 3-aminophenol was added into the solution. The mixture was stirred at room temperature for another 1 h before adding the corresponding amount of formaldehyde solution (38 wt%) to start the polymerization for 24 h. Then the previous mixture solution was transferred into an autoclave and sealed to heat at 180 °C for 6 h. The obtained brown black particles were filtered and washed with deionized water and ethanol to remove residues, and evaporated at 60 °C under vacuum. The carbonization was carried out in a tubular furnace under nitrogen atmosphere in two steps: the sample was heated to 300 °C for 1 h (heating rate: 1 °C min^−1^) and 1000 °C for 2 h (heating rate: 5 °C min^−1^). The resulted composites (named Cu_2_O@C@N) were immersed in concentrated 30 ml of hydrochloric acid (37 wt%), and 30 ml of hydrogen peroxide solution (10 wt%) added dropwise into the solution, then stirred for 8 h at room temperature to dissolve the Cu_2_O templates, this process was repeated at least three times until the copper ions were completely removed. The NHCSs materials were collected by centrifugation, washed with distilled water and dried at 60 °C for 5 h under vacuum. Finally, the obtained samples were denoted as S1. For comparison, we prepared three additional sets of samples with different carbon shell thicknesses. (denoted as S2, S3 and S4, respectively. ESI, Table S1†).

### 2.3. Characterization

The morphology of samples were investigated by field emission scanning electron microscope (FESEM, Quanta 200 FEG) and transmission electron microscope (TEM, JEM2100). The crystallographic structures of the samples were analyzed by X-ray diffraction (XRD, X Pert PRO) equipped with Cu-Kα radiation (*λ* = 0.154 nm) source at scanning speed of 10° min^−1^ in the range of 10–80°and Raman spectrum (DXR-Raman) under *λ*_exc_ = 514 nm laser excitation. The pore structure of the materials was examined by N_2_ adsorption–desorption at 77 K (Micrometrics, ASAP 2020). The BET surface area (*S*_BET_) of samples was calculated according to the BET (Brunauer–Emmett–Teller). The chemical bonding state of carbon, oxygen and nitrogen elements in NHCSs were studied by X-ray photoelectron spectroscopy (XPS, Thermo Scientific ESCALAB250Xi).

### 2.4. Electrochemical measurement

The working electrodes were prepared by mixing the NHCSs with 10 wt% of polytetrafluoroethylene (PTFE) and acetylene black (the mass ratio of 8 : 1 : 1) in *N*-methyl-2-pyrrolidone to form a homogenous slurry. The slurry was coated on nickel foam (1 cm × 2 cm) with a surface area of 1 cm^2^, the electrodes were dried in vacuum at 120 °C for 12 h and pressed under a pressure of 10 MPa for 30 s. The loading active material was in the range of 3.0–6.0 mg cm^−2^. All electrochemical behaviour of the working electrodes were investigated in a three-electrode system with 6 M KOH aqueous solution as the electrolyte. The saturated calomel electrode (SCE) and platinum were served as reference electrode and counter electrode, respectively. Cyclic voltammetry (CV), galvanostatic charge–discharge (GCD) and life cycle test were conducted on a CHI660E electrochemical workstation (Shanghai Chenhua, China). The range of CV test varied from −1.0 to 0 V with the different scan rates of 10–100 mV s^−1^ and the current density of GCD were from 0.5 to 20 A g^−1^. The specific capacitance was calculated from GCD according to equation:^28^*C*_g_ = *I*Δ*t*/Δ*Vm*;where *C*_g_ is the gravimetric-specific capacitance, *I* is the discharge current (A); Δ*t* is the discharge time (s), Δ*V* is the potential window (V) and *m* is the mass of the active material on the working electrode (g), respectively.

## 3. Result and discussion

**Fig. 1:**
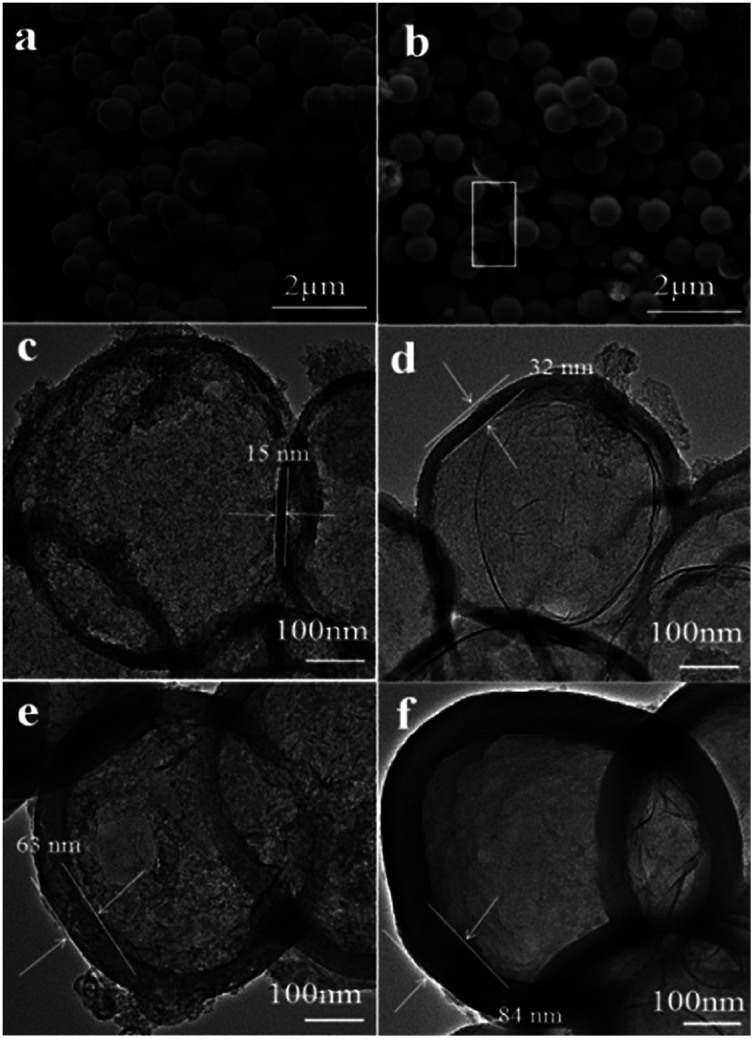
SEM images of (a) Cu_2_O microspheres and nitrogen-doped hollow carbon spheres (b) S1; TEM images of NHCSs with different thickness of carbon shell (c) S1, 25 nm (d) S2, 32 nm (e) S3, 63 nm and (f) S4, 84 nm.

The morphology and microstructure of the prepared samples were characterized by scanning electron microscope (SEM) and transmission electron microscope (TEM). As can be seen in Fig. 1a, the Cu_2_O microspheres present fully composed of spherical particles with the diameter of 600–700 nm, and these microspheres have rough surfaces. After self-polymerization of 3-aminophenol formaldehyde resin, followed by carbonization and etching template, all obtained NHCSs mainly inherit the spherical structure with smooth surfaces, and the diameters of the carbon spheres are almost unchanged compared with the Cu_2_O templates. Fig. S1a–d (ESI†) clearly show the morphology evolution of NHCSs with different carbon content. Some cracked NHCSs observed suggest their hollow structure (as shown in white rectangle of Fig. 1b). It should be mentioned that using the prepared Cu_2_O microspheres as cores, carbon coating can be achieved on these microspheres through *in situ* polymerization of 3-aminophenol formaldehyde resin following by a carbonization process to produce composites with hollow structures. When the amount of 3-aminophenol gradually increases, the thickness of NHCSs display monotonic increase with more 3-aminophenol and formaldehyde applied in the experiments, and the hollow structure of the NHCS with the larger thickness is also more and more difficult to be destroyed during the etching template process. (Fig. S1, ESI†). According to the above results, we can conclude that the content of 3-aminophenol and formaldehyde in synthesis system plays a key role on the morphology and the thickness of the NHCSs. Fig. 1c–f show the TEM images of all the samples, those images further reveal the hollow spherical morphology of the NHCSs. In addition, these spherical NHCSs went through a slight deformation, this might be attributed to the fact that cuprous oxide loses one oxygen atom after being reduced by carbon, resulting in a change in the spherical structure of the hard tin plating agent, thereby slightly deforming the spherical morphology of the NHCSs. Furthermore, it is obviously that the variation of the content of carbon source affects the uniformity and thickness of the NHCSs remarkably. From these TEM images, we can see that those hollow structured NHCSs can be fabricated with the diameter of >600 nm and the thicknesses of carbon shells for S1, S2, S3 and S4 are about 15 nm, 32 nm, 63 nm and 84 nm, respectively, while the carbon shell of S1 collapses after removing the Cu_2_O cores due to its thinnest carbon shell. These above results further indicate that the carbon shell can be successfully prepared on the cores by properly controlling the experimental conditions and the relative ratio of 3-aminophenol to Cu_2_O microspheres, and more importantly, the shell thickness can be adjusted at the nanoscale.

The N_2_ adsorption/desorption characterization was further carried out to evaluate the pore structure of NHCSs. The adsorption/desorption isotherm and the corresponding pore size distribution are shown in the ESI (Fig. S2a and S2b),† respectively. All samples exhibit typical type IV isotherms according to the IUPAC classification, indicating the assembly of micro and mesoporous.^29^ The pore size distribution was analyzed using the Barrette–Joynere–Halenda (BJH) method and the distribution curves of the S1, S2, S3 and S4 show that their average pore sizes are around 1.3 nm (Fig. S2b, ESI†). A summary of the surface area, average pore size and total volume is recorded in Table 1, The BET surface areas of S1, S2, S3 and S4 are 112.36, 74.05, 25.65, and 18.93 m^2^ g^−1^ and the pore volumes are 0.34, 0.24, 0.08 and 0.07 cm^3^ g^−1^, respectively, which is basically negatively correlated with the thickness of carbon shell of NHCSs. Notably, the microporous and mesoporous assembly structures of NHCSs could provide a huge amount of “ion-adsorption sites” for the formation of electric double layer, showing a promise to explore the synthesized NHCSs as electrode materials for advanced supercapacitor.

**Table 1:** Texture properties of NHCSs

Sample	*S*_BET_ (m^2^ g^−1^)	*V*_p_ a (cm^3^ g^−1^)	Pore size^b^ (nm)	C^c^ (wt%)	O^c^ (wt%)	N^c^ (wt%)
S1	112.36	0.34	1.21	86.98	7.93	5.09
S2	74.05	0.24	1.34	86.72	8.27	5.01
S3	25.65	0.08	1.27	87.69	7.34	4.97
S4	18.93	0.07	1.49	87.26	7.79	4.95

a Total pore volume from N_2_ adsorbed at *p*/*p*_0_ = 0.995.

b Maximum of pore size distribution.

c C, N and O, the content of carbon, oxide and nitrogen tested by XPS.

**Fig. 2:**
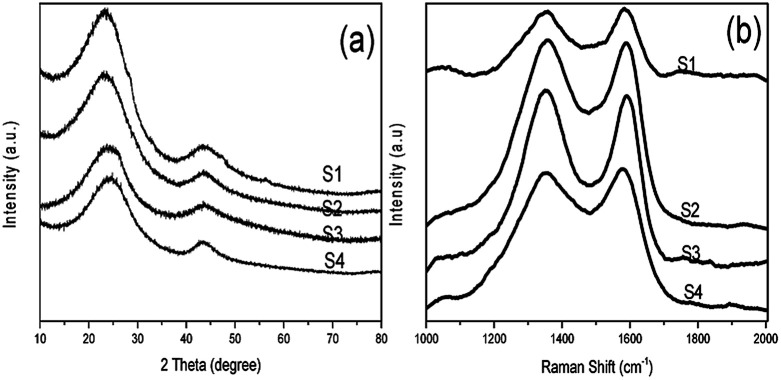
(a) XRD pattern and (b) Raman spectra of NHCSs (S1, S2, S3 and S4).

The carbon state in NHCSs was identified by the X-ray diffraction (XRD) and Raman spectroscopy. As shown in Fig. 2a, all samples exhibit two typical broadened diffraction peaks at about 2*θ* = 26° and 43°, which can be approximately indexed as (002) and (100) reflection of standard graphite, respectively.^25,30,31^ The pronounced intensity of the wide (002) diffraction peak attributing to stacks of parallel layer planes indicates certain graphitizing degree of the as fabricated NHCSs. With the decrease of the thickness of carbon shell, the (002) peak gradually has a slight upward trend, indicating that the NHCSs has the tendency of graphitization. By comparing the XRD pattern of Cu_2_O and Cu_2_O@C@N (as shown in Fig. S2, ESI†), there is no peak which is ascribed to copper (or cuprous oxide) appeared in the pattern, implying the complete removal of copper and cuprous oxide species during the templates etching. The Raman spectrum of the NHCSs depicted in Fig. 2b exhibits two peaks at about 1350 cm^−1^ (D band) and 1580 cm^−1^ (G band). The D band is considered to the crystal defects and disordered structures of carbon materials, while the G band is attributed to the phonon mode with *E*_2g_ symmetry of graphite.^31,32^ By calculating the relative intensity of the D band and the G band (*I*_D_/*I*_G_), the graphitic degree of the carbon materials can be determined.^33^ The *I*_D_/*I*_G_ intensity ratios of S1, S2, S3 and S4 are 0.98, 0.99, 1.01 and 1.02, respectively, indicating the high degree of defects for obtained NHCSs, which is good agreement with the above XRD result.

**Fig. 3:**
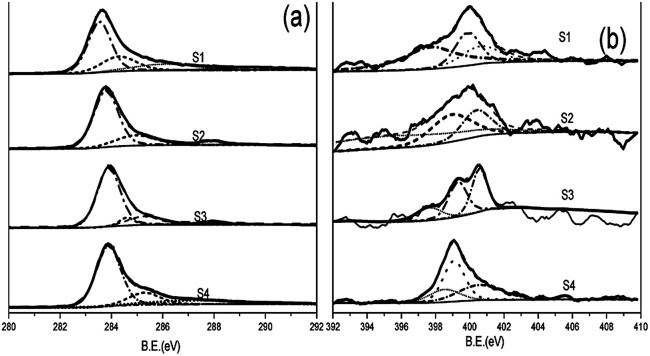
XPS spectrum of (a) C 1s spectrum and (b) N 1s spectrum of NHCSs.

The chemical composition and electronic state of the materials were analyzed by X-ray photoelectron spectroscopy (XPS). As is shown in the ESI (Fig. S3),† the XPS spectrum of the obtained NHCSs shows the presence of typical C 1s, N 1s and O 1s peaks, which clearly indicates that the N atoms were successfully doped into the carbon structure in NHCSs. The surface chemistry of NHCSs is dominated by carbon, oxygen and nitrogen, and the content of nitrogen in materials are 5.09, 5.01 4.97 and 4.95 wt%, listed in Table 1. Fig. 3a presents the C 1s spectrum of NHCSs, which can be decomposed into the following four Gaussian peaks: 284.6 eV (sp^2^-hybridized graphitic C, C–I), 285.9 eV (sp^2^ C–N and C–O in phenols and ethers, C-II), 286.8 eV (C=O, C-III), and 288.7 eV (O–C=O, C-IV).^33,34^ Gaussian Fitting of N 1s spectra indicates pyridinic-N, pyrrolic-N, quaternary-N, attributable to binding energies of 398.1 eV, 399.5 eV and 400.7 eV,^35,36^ respectively (Fig. 3b). As reported previously, pyridinic-N and pyrrolic-N play a key role in increasing the specific capacitance of N-doped materials due to their pseudo-capacitive contribution, whereas quaternary-N can increase the conductivity of the material and thus improve the capacitance performance.^37,38^ In addition, the suitable nitrogen and oxygen doping can increase the surface wettability with aqueous electrolyte and thus reduce the resistance of electrode materials.^34,39,40^

**Fig. 4:**
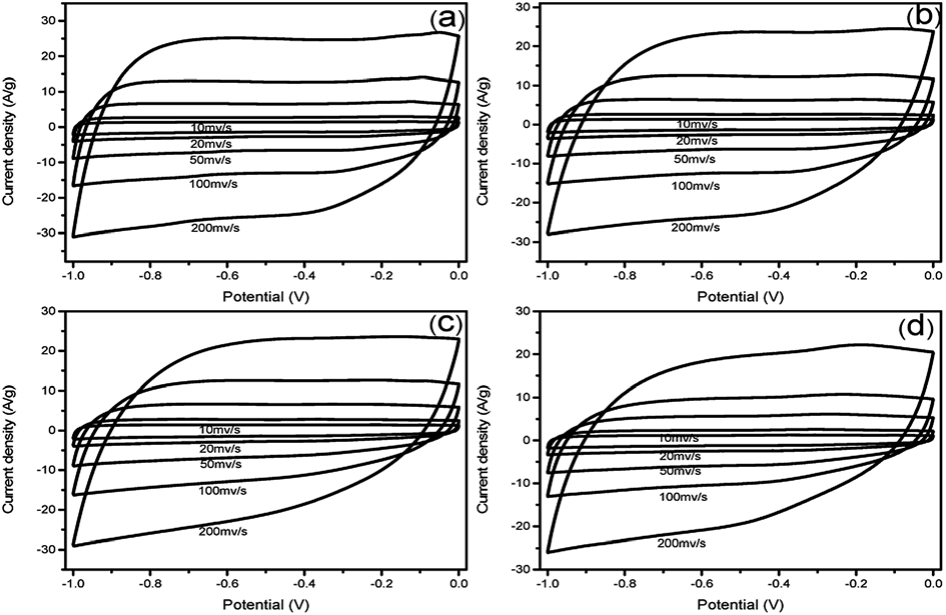
CV curves of NHCSs with different carbon shell thickness (a) S1, (b) S2, (c) S3 and (d) S4 at different scan rates range from 10 to 200 mV s^−1^ in 6 M KOH electrolyte.

To evaluate the electrochemical properties of the prepared N-doped carbon materials for supercapacitor electrodes, the samples were characterized by cyclic voltammetry (CV) and galvanostatic charge–discharge (GCD) measurements within the potential window of −1.0 to 0 V in 6 M KOH aqueous solution. Fig. 4 shows the CV curves of NHCSs electrodes at scan rates of 10–200 mV s^−1^. All the CV curves display approximately rectangular shapes, indicating the typical characteristic of electrical double layer capacitor (EDLC).^34^ From the results of the curves, we can see that the S1 gives the largest quasi-rectangular area at the same scan rate,namely, the highest specific capacitance, which could be ascribed to the ultrafast electron transfer and electrolyte transportation rates, which is caused by the specific surface area and ultra-thin carbon layer of S1.

**Fig. 5:**
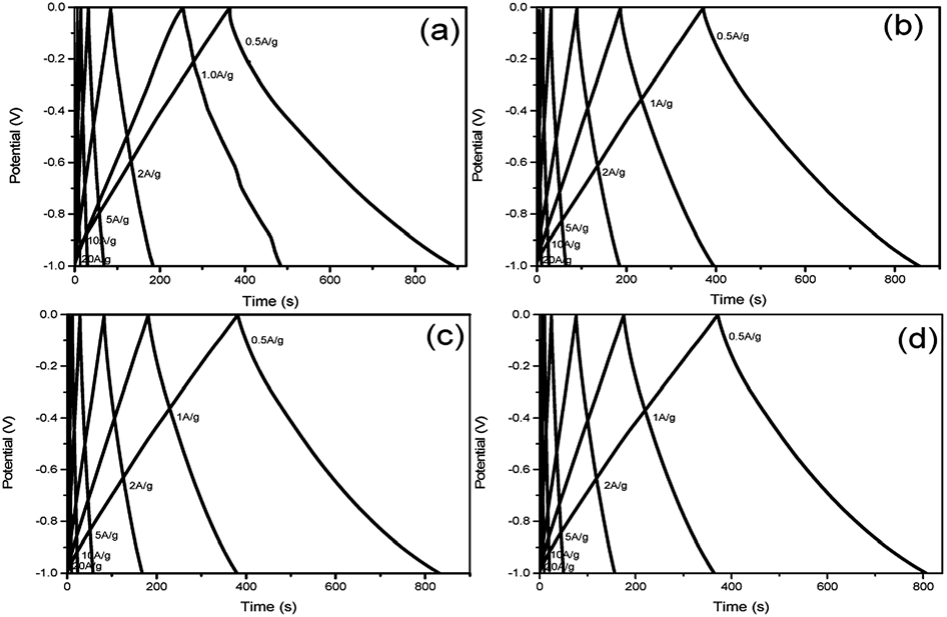
Galvanostatic charge–discharge curves of NHCSs (a) S1, (b) S2, (c) S3 and (d) S4 at various current densities (0.5–20 A g^−1^).

The GCD curves of at various current densities (0.5, 1, 2, 5, 10, 20 A g^−1^) in 6 M KOH aqueous solution display in Fig. 5a–d respectively. Basically, all these curves emerge in a nearly symmetric triangle shape along with a slight distortion, furthermore confirming the typical characteristic of EDLC. According to the formula,^28^ the specific capacitance of S1 at 0.5 A g^−1^ is 263.6 F g^−1^, higher than that of S2 (241.1 F g^−1^), S3 (225.0 F g^−1^) and S4 (217.7 F g^−1^). Through the comparison of these four samples, it can be clearly seen that S1 shows the highest specific capacitance, indicating that the specific capacitance of NHCSs is negatively correlated with the thickness of carbon shell. The optimal specific capacitance of S1 may come from its highest specific surface area, the thinnest carbon shell and the largest pore volume pore size, which facilitates the formation of electric bilayers. In addition, nitrogen doped in the carbon structure can also create pseudo-capacitance and improve the surface wettability.

**Fig. 6:**
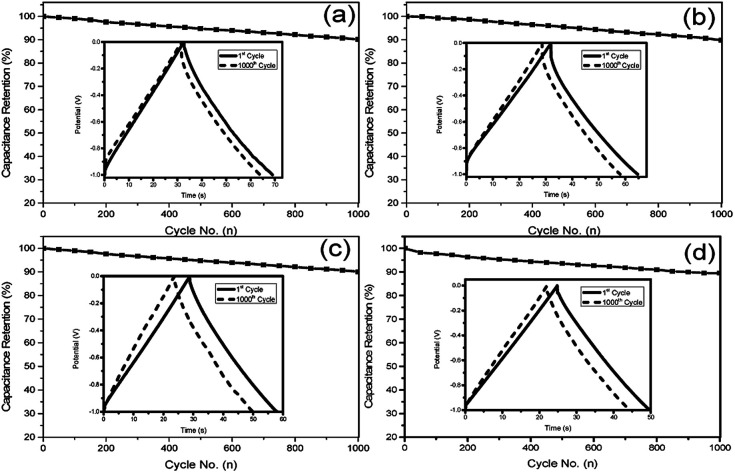
Cycling stability measured for NHCSs (a) S1, (b) S2, (c) S3 and (d) S4 at 5 A g^−1^ over 1000 cycles in 6 M KOH electrolyte.

The specific capacitance values of all samples decrease with the increase of charge/discharge current densities (as shown in Fig. S4, ESI†), which is due to the satirical limitations for partial ions penetrating into micropores at higher current density, whereas smoothly diffusing at lower current density.^41^ Obviously, the obtained S1 exhibits better electrochemical performance than others, which can be attributed to two reasons. Firstly, the thin carbon shell and the high *S*_BET_ can provide shorter ion transport distances and more electrochemically active sites for ion accommodation and fast ion migration. Secondly, the high amount of nitrogen doping and the tendency of graphitization can provide S1 with pseudo-capacitance and increase its conductivity. Both of the two factors generate a synergistic effect, which is advantageous for the high capacitance of S1. The cycling performance of supercapacitors is another crucial property for practical applications, and the cycling stability of NHCSs electrode was estimated by galvanostatic charge–discharge profiles for 1000 cycles at current density of 5 A g^−1^. As depicted in Fig. 6a–d, the specific capacitances of those four samples all remain at about 90%, indicating that they all demonstrate good cycle performance. In addition, taking S1 with the best electrochemical performance among all samples, we also investigated the morphological changes of NHCSs after long time charging and discharging. Fig. S6† shows the SEM image of S1 after the long cycle test. The image shows that a small amount of carbon spherical morphology has been damaged, which could be ascribed to one of the reasons for its good chemical cycle stability.

## 4. Conclusions

In summary, using Cu_2_O microspheres as recycle hard template, NHCSs with tunable shell thickness have been successfully synthesized through *in situ* polymerization of 3-aminophenol formaldehyde resin followed by high-temperature carbonization and templates etching. The prepared NHCSs combine the characteristics of special morphology, controllable shell thickness, unique porosity and nitrogen functional groups. Electrochemical tests showed that S1 with the thinnest carbon shell exhibited outstanding supercapacitor performance with good capacitance (263.6 F g^−1^ at 0.5 A g^−1^), ultrahigh-rate performance (122.0 F g^−1^ at 20 A g^−1^) and excellent long-term cycling stability (90.2% retention after 1000 cycles at 5 A g^−1^) in 6 M KOH aqueous electrolyte. Based on the unique characteristics of the NHCSs, we believe that the well-developed NHCSs with high electrochemical performance provide new opportunities for energy storage and energy conversion applications, such as supercapacitors and lithium–sulfur batteries.^42^

## Conflicts of interest

There are no conflicts to declare.

## Supplementary Material

RA-010-D0RA02935A-s001

## References

[cit1] Zhang Z., Qin M., Jia B., Zhang H., Wu H., Qu X. (2017). Facile synthesis of novel bowl-like hollow carbon spheres by the combination of hydrothermal carbonization and soft templating. Chem. Commun..

[cit2] Hu Y., Jensen J. O., Zhang W., Cleemann L. N., Xing W., Bjerrum N. J., Li Q. (2014). Hollow spheres of iron carbide nanoparticles encased in graphitic layers as oxygen reduction catalysts. Angew. Chem., Int. Ed..

[cit3] Shao Q., Tang J., Lin Y., Zhang F., Yuan J., Zhang H., Qin L. C. (2013). Synthesis and characterization of graphene hollow spheres for application in supercapacitors. J. Mater. Chem. A.

[cit4] Zhu Y., Wang F., Zhang C., Du J. (2014). Preparation and mechanism insight of nuclear envelope-like polymer vesicles for facile loading of biomacromolecules and enhanced biocatalytic activity. ACS Nano.

[cit5] Ikeda S., Ishino S., Harada T., Okamoto N., Sakata T., Mori H., Matsumura M. (2006). Ligand-Free Platinum Nanoparticles Encapsulated in a Hollow Porous Carbon Shell as a Highly Active Heterogeneous Hydrogenation Catalyst. Angew. Chem..

[cit6] Schaefer Z. L., Gross M. L., Hickner M. A., Schaak R. E. (2010). Uniform Hollow Carbon Shells: Nanostructured Graphitic Supports for Improved Oxygen-Reduction Catalysis. Angew. Chem., Int. Ed..

[cit7] Chai G. S., Yoon S. B., Kim J. H., Yu J. S. (2004). Spherical carbon capsules with hollow macroporous core and mesoporous shell structures as a highly efficient catalyst support in the direct methanol fuel cell. Chem. Commun..

[cit8] Fang X., Zang J., Wang X., Zheng M. S., Zheng N. (2014). A multiple coating route to hollow carbon spheres with foam-like shells and their applications in supercapacitor and confined catalysis. J. Mater. Chem. A.

[cit9] Fang B., Kim J. H., Kim M. S., Bonakdarpour A., Lam A., Wilkinson D. P., Yu J. S. (2012). Fabrication of hollow core carbon spheres with hierarchical nanoarchitecture for ultrahigh electrical charge storage. J. Mater. Chem..

[cit10] Zhu C., Wang M., Yang G., Lu T., Pan L. (2017). N, P dual-doped hollow carbon spheres for high-performance supercapacitors. J. Solid State Electrochem..

[cit11] Liu L., Xu S. D., Yu Q., Wang F. Y., Zhu H. L., Zhang R. L., Liu X. (2016). Nitrogen-doped hollow carbon spheres with a wrinkled surface: their one-pot carbonization synthesis and supercapacitor properties. Chem. Commun..

[cit12] Thirumal V., Pandurangan A., Jayavel R., Ilangovan R. (2016). Synthesis and characterization of boron doped graphene nanosheets for supercapacitor applications. Synth. Met..

[cit13] Huang Y., Candelaria S. L., Li Y., Li Z., Tian J., Zhang L., Cao G. (2014). Sulfurized activated carbon for high energy density supercapacitors. J. Power Sources.

[cit14] Gu W., Sevilla M., Magasinski A., Fuertes A. B., Yushin G. (2013). Sulfur-containing activated carbons with greatly reduced content of bottle neck pores for double-layer capacitors: a case study for pseudocapacitance detection. Energy Environ. Sci..

[cit15] Inagaki M., Toyoda M., Soneda Y., Morishita T. (2018). Nitrogen-doped carbon materials. Carbon.

[cit16] You J., Dou L., Yoshimura K., Kato T., Ohya K., Moriarty T., Yang Y. (2013). A polymer tandem solar cell with 10.6% power conversion efficiency. Nat. Commun..

[cit17] Cai T., Xing W., Liu Z., Zeng J., Xue Q., Qiao S., Yan Z. (2015). Superhigh-rate capacitive performance of heteroatoms-doped double shell hollow carbon spheres. Carbon.

[cit18] Yuan C., Liu X., Jia M., Luo Z., Yao J. (2015). Facile preparation of N-and O-doped hollow carbon spheres derived from poly (o-phenylenediamine) for supercapacitors. J. Mater. Chem. A.

[cit19] Yang T., Liu J., Zhou R., Chen Z., Xu H., Qiao S. Z., Monteiro M. J. (2014). N-doped mesoporous carbon spheres as the oxygen reduction reaction catalysts. J. Mater. Chem. A.

[cit20] Liu D., Tufa L. T., Lee J. (2019). N-doped microporous carbon hollow spheres with precisely controlled architectures for supercapacitor. Electrochim. Acta.

[cit21] Xia Y., Yang Z., Mokaya R. (2004). Mesostructured hollow spheres of graphitic N-doped carbon nanocast from spherical mesoporous silica. J. Phys. Chem. B.

[cit22] Li N., Wang Z., Zhao K., Shi Z., Gu Z., Xu S. (2010). Large scale synthesis of N-doped multi-layered graphene sheets by simple arc-discharge method. Carbon.

[cit23] Liu J., Yang T., Wang D. W., Lu G. Q. M., Zhao D., Qiao S. Z. (2013). A facile soft-template synthesis of mesoporous polymeric and carbonaceous nanospheres. Nat. Commun..

[cit24] Chen A., Xia K., Zhang L., Yu Y., Li Y., Sun H., Li S. (2016). Fabrication of nitrogen-doped hollow mesoporous spherical carbon capsules for supercapacitors. Langmuir.

[cit25] Qu Y., Zhang Z., Du K., Chen W., Lai Y., Liu Y., Li J. (2016). Synthesis of nitrogen-containing hollow carbon microspheres by a modified template method as anodes for advanced sodium-ion batteries. Carbon.

[cit26] Han J., Xu G., Ding B., Pan J., Dou H., MacFarlane D. R. (2014). Porous nitrogen-doped hollow carbon spheres derived from polyaniline for high performance supercapacitors. J. Mater. Chem. A.

[cit27] Tang J., Liu J., Salunkhe R. R., Wang T., Yamauchi Y. (2016). Nitrogen-doped hollow carbon spheres with large mesoporous shells engineered from diblock copolymer micelles. Chem. Commun..

[cit28] Jiang L., Sheng L., Long C., Fan Z. (2015). Densely packed graphene nanomesh-carbon nanotube hybrid film for ultra-high volumetric performance supercapacitors. Nano Energy.

[cit29] Du J., Liu L., Yu Y. (2019). *et al.*, Tuning Confined Nanospace for Preparation of N-doped Hollow Carbon Spheres for High Performance Supercapacitors. ChemSusChem.

[cit30] Hou L., Lian L., Li D., Pang G., Li J., Zhang X., Yuan C. (2013). Mesoporous N-containing carbon nanosheets towards high-performance electrochemical capacitors. Carbon.

[cit31] Quan B., Jin A., Yu S. H., Kang S. M., Jeong J., Abruña H. D., Sung Y. E. (2018). Solvothermal-Derived S-Doped Graphene as an Anode Material
for Sodium-Ion Batteries. Adv. Sci..

[cit32] Sadezky A., Muckenhuber H., Grothe H., Niessner R., Pöschl U. (2005). Raman microspectroscopy of soot and related carbonaceous materials: spectral analysis and structural information. Carbon.

[cit33] Li M., Zhang Y., Yang L., Liu Y., Yao J. (2015). Hollow melamine resin-based carbon spheres/graphene composite with excellent performance for supercapacitors. Electrochim. Acta.

[cit34] Zhang D., Hao Y., Zheng L., Ma Y., Feng H., Luo H. (2013). Nitrogen and sulfur co-doped ordered mesoporous carbon with enhanced electrochemical capacitance performance. J. Mater. Chem. A.

[cit35] Sun Z., Masa J., Weide P., Fairclough S. M., Robertson A. W., Ebbinghaus P., Schuhmann W. (2015). High-quality functionalized few-layer graphene: facile fabrication and doping with nitrogen as a metal-free catalyst for the oxygen reduction reaction. J. Mater. Chem. A.

[cit36] Sun Z., Shen S., Ma L., Mao D., Lu G. (2016). Controlled synthesis of N-doped carbon spheres with different morphologies for supercapacitors. RSC Adv..

[cit37] Sun H., Zhu Y., Yang B., Wang Y., Wu Y., Du J. (2016). Template-free fabrication of nitrogen-doped hollow carbon spheres for high-performance supercapacitors based on a scalable homopolymer vesicle. J. Mater. Chem. A.

[cit38] Zhong H., Zhang H., Liu S., Deng C., Wang M. (2013). Nitrogen-Enriched Carbon from Melamine Resins with Superior Oxygen Reduction Reaction Activity. ChemSusChem.

[cit39] Tian H., Lin Z., Xu F., Zheng J., Zhuang X., Mai Y., Feng X. (2016). Quantitative Control of Pore Size of Mesoporous Carbon Nanospheres through the Self-Assembly of Diblock Copolymer Micelles in Solution. Small.

[cit40] Wickramaratne N. P., Xu J., Wang M., Zhu L., Dai L., Jaroniec M. (2014). Nitrogen enriched porous carbon spheres: attractive materials for supercapacitor electrodes and CO_2_ adsorption. Chem. Mater..

[cit41] Ma X., Liu M., Gan L., Zhao Y., Chen L. (2013). Synthesis of micro-and mesoporous carbon spheres for supercapacitor electrode. J. Solid State Electrochem..

[cit42] Peng Y., Zhang Y., Huang J., Wang Y., Li H., Hwang B. J., Zhao J. (2017). Nitrogen and oxygen dual-doped hollow carbon nanospheres derived from catechol/polyamine as sulfur hosts for advanced lithium sulfur batteries. Carbon.

