# Supplementary material for: Nitrogen-doped hollow carbon spheres with tunable shell thickness for high-performance supercapacitors
Source: RSC Adv. 2020 Jul 14;10(44):26546–52. doi: 10.1039/d0ra02935a (PMC9055434; doi:10.1039/d0ra02935a)
Supplement: RA-010-D0RA02935A-s001 [file RA-010-D0RA02935A-s001.pdf]

Electronic Supplementary Material (ESI) for RSC Advances.
This journal is © The Royal Society of Chemistry 2020

Supporting information

Nitrogen-doped Hollow Carbon Spheres with Tunable Shell Thickness for High-Performance Supercapacitor

Dawei Zhang, Shaodian Shen, Xiuzhen Xiao, Dongsen Mao, Baoman Yan

†Research Institute of Applied Catalysis, School of Chemical and Environmental Engineering, Shanghai Institute of Technology, Shanghai, 201418, P. R.China

Table S1 The details of the materials formulations for NHCSs

Sample no.	Cu_2O /g	3-aminophenol /g	formaldehyde /ml	Shell thickness /nm
S1	1.5	0.1	0.14	15
S2	1.0	0.1	0.14	32
S3	1.0	0.2	0.28	63
S4	1.0	0.3	0.42	84

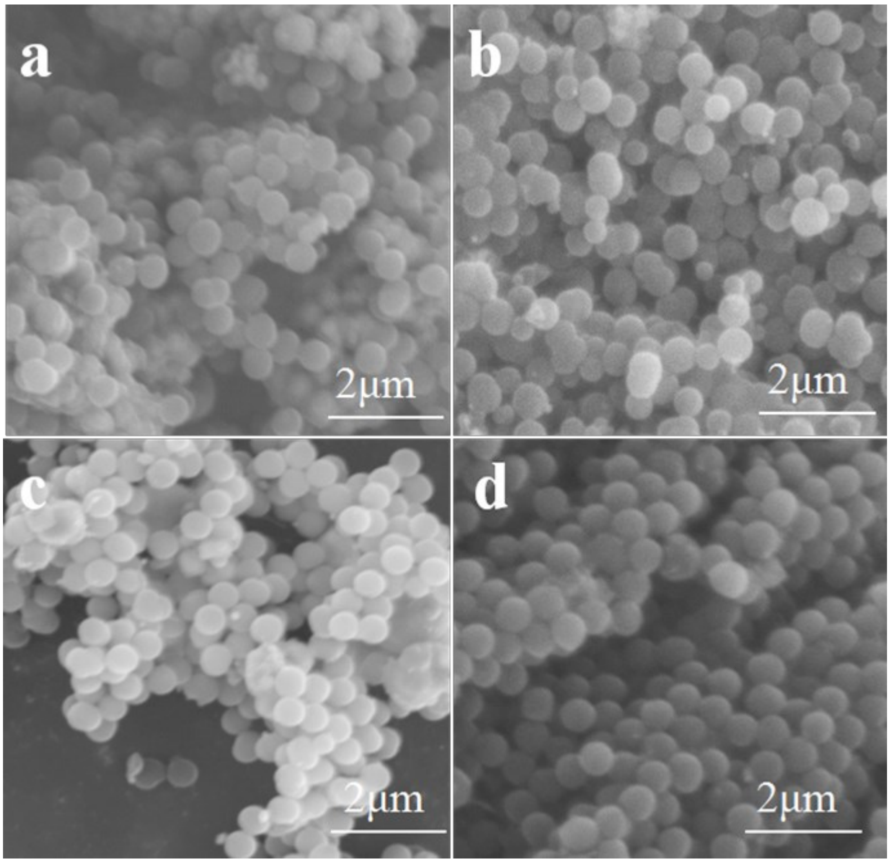

Figure S1 SEM images of NHCSs : (a) S1, (b) S2 , (c) S3 and (d) S4.

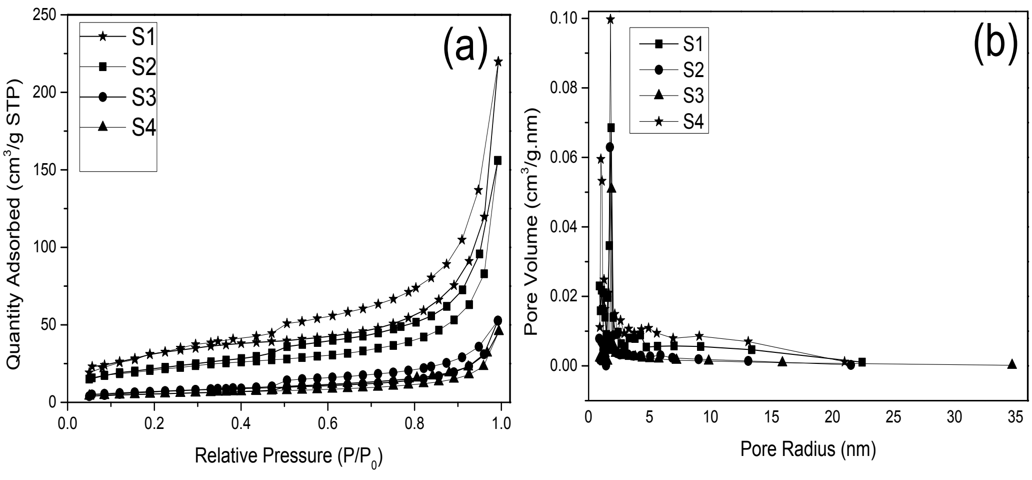

Figure S2. N_2 adsorbed/desorption isotherms (a) and pore size distribution of NHCSs.

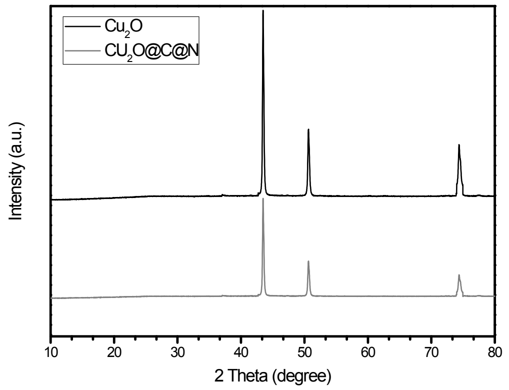

Figure S3. XRD pattern of Cu_2O microspheres and Cu_2O@C@N (before template etching to remove Cu).

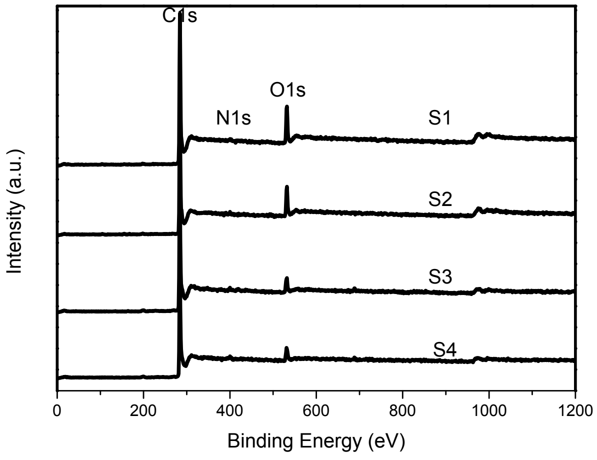

Figure S4. XPS spectrum of NHCSs:

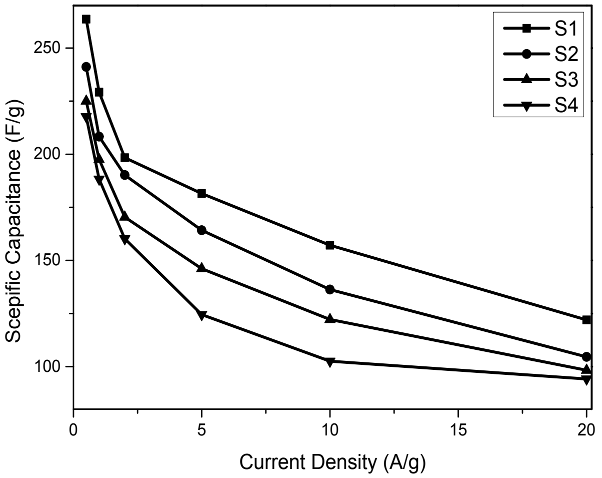

Figure S5. The relationship between specific capacitance values and current densities.(0.5, 1, 2, 5, 10, 20 A g^{-1})

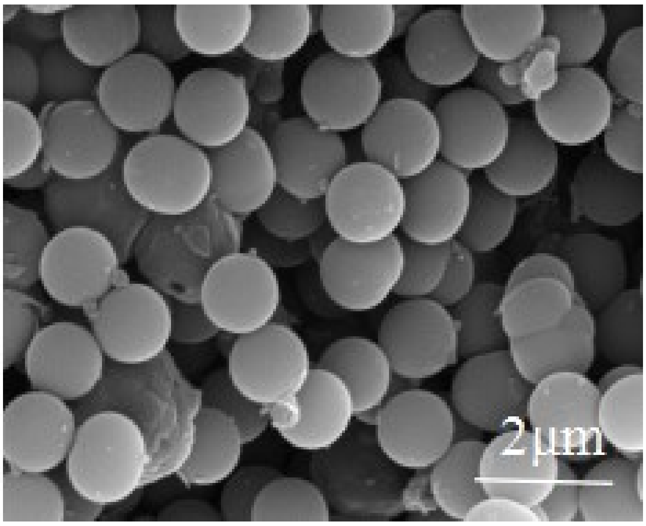

Figure S6. SEM images of S1 estimated by galvanostatic charge-discharge profiles for 1000 cycles at current density of 5 A g^{-1}.
